# Considerações Metodológicas sobre Disparidades Étnico-Raciais e Controle da Hipertensão Arterial

**DOI:** 10.36660/abc.20260128

**Published:** 2026-06-23

**Authors:** Maikon Lucian Madeira Quarti, Arina Peixoto Nobre, Gabriel Kaleb Martins

**Affiliations:** 1 Hospital e Maternidade Marieta Konder Bornhausen Serviço de Cirurgia Cardiovascular Itajaí SC Brasil Serviço de Cirurgia Cardiovascular, Hospital e Maternidade Marieta Konder Bornhausen, Itajaí, SC – Brasil; 2 Universidade de Fortaleza Faculdade de Medicina Fortaleza CE Brasil Faculdade de Medicina, Universidade de Fortaleza, Fortaleza, CE – Brasil; 3 Instituto de Educação Médica Faculdade de Medicina Alagoinhas BA Brasil Faculdade de Medicina, Instituto de Educação Médica, Alagoinhas, BA – Brasil

**Keywords:** Hipertensão, Etnicidade, Metodologia como Assunto, Disparidades nos Níveis de Saúde

**Prezado Editor,**

Lemos com interesse o Artigo Original de Euzébio et al.,^1^"Comparação dos Valores de Pressão Arterial e dos Medicamentos Anti-Hipertensivos Utilizados por Brasileiros não Afrodescendentes e Afrodescendentes com Hipertensão", publicado no *Arquivos Brasileiros de Cardiologia*. O tema é relevante diante das desigualdades étnico-raciais nos desfechos cardiovasculares no Brasil. Contudo, alguns aspectos metodológicos e analíticos merecem consideração.

Inicialmente, a definição operacional de raça/cor exige cautela. A dicotomização entre "afrodescendentes" e "não afrodescendentes", ao agrupar indivíduos autodeclarados como brancos, pardos e asiáticos em um único grupo, não reflete a complexidade da classificação racial brasileira segundo o IBGE, podendo introduzir viés de classificação e diluir heterogeneidades social e clinicamente relevantes.^2^

No que se refere à metodologia estatística, a escolha entre testes paramétricos e não paramétricos parece decorrer de avaliação *post hoc* das premissas estatísticas, em vez de estratégia analítica definida *a priori*.

Adicionalmente, os modelos de regressão se concentram no uso de betabloqueadores, sem ajuste adequado para gravidade da doença ou análise formal de interação com raça/cor, o que limita a interpretação clínica dos achados à luz das diretrizes contemporâneas.

Por fim, embora o estudo utilize dados de um registro nacional multicêntrico, não é apresentado um plano analítico multivariado pré-especificado, baseando-se em comparações univariadas ou ajustes limitados, sem incorporação simultânea de variáveis fundamentais, como comorbidades, intensidade terapêutica e contexto assistencial. Evidências populacionais demonstram que fatores estruturais e do sistema de saúde impactam os desfechos cardiovasculares mais do que variáveis demográficas isoladas.^3^

Nesse contexto, apesar das diferenças clínicas observadas, o estudo não aprofunda a análise dos determinantes sociais da saúde, limitando a interpretação dos achados.^4^ Ademais, o período de coleta dos dados (2013–2015) impõe restrições à extrapolação dos resultados para a prática clínica atual.

Cumprimentamos os autores pela iniciativa e esperamos que as considerações contribuam para o debate científico sobre desigualdades raciais e controle da hipertensão.
