# Supplementary material for: Considerações Metodológicas sobre Disparidades Étnico-Raciais e Controle da Hipertensão Arterial
Source: Arq Bras Cardiol. 2026 Jun 23;123(5):e20260128. [Article in Portuguese] doi: 10.36660/abc.20260128 (PMC13399037; doi:10.36660/abc.20260128)
Supplement: Translation

# Translation: Methodological Considerations on Ethno-Racial Disparities and Blood Pressure Control

**DOI:** 10.36660/abc.20260128i

**Authors:** Maikon Lucian Madeira Quarti, Arina Peixoto Nobre, Gabriel Kaleb Martins

**Affiliations:** 1 Itajaí SC Brazil Serviço de Cirurgia Cardiovascular, Hospital e Maternidade Marieta Konder Bornhausen, Itajaí, SC – Brazil; 2 Fortaleza CE Brazil Faculdade de Medicina, Universidade de Fortaleza, Fortaleza, CE – Brazil; 3 Alagoinhas BA Brazil Faculdade de Medicina, Instituto de Educação Médica, Alagoinhas, BA – Brazil

**Keywords:** Hypertension, Ethnicity, Methodology as a Subject, Health Status Disparities

**Dear Editor,**

We read with interest the Original Article by Euzébio et al.,^1^ "Comparison of Blood Pressure Values and Antihypertensive Medications Used in Non-Afro-Descendant and Afro-Descendant Brazilians with Hypertension", published in the *Arquivos Brasileiros de Cardiologia*. The topic is relevant given the ethnic-racial inequalities in cardiovascular outcomes in Brazil. However, some methodological and analytical aspects deserve consideration.

Initially, the operational definition of race/color requires caution. The dichotomy between "Afro-descendants" and "non-Afro-descendants," by grouping individuals who self-identify as white, brown, and Asian into a single group, does not reflect the complexity of Brazilian racial classification according to the IBGE (Brazilian Institute of Geography and Statistics), and may introduce classification bias and dilute socially and clinically relevant heterogeneities.^2^

Concerning statistical methodology, the choice between parametric and non-parametric tests seems to stem from a post-hoc evaluation of statistical assumptions, rather than from an analytical strategy defined a priori.

Additionally, the regression models focus on the use of beta-blockers, without adequate adjustment for disease severity or formal analysis of interaction with race/color, which limits the clinical interpretation of the findings in light of contemporary guidelines.

Finally, although the study uses data from a multicenter national registry, it does not present a pre-specified multivariate analytical plan, relying instead on univariate comparisons or limited adjustments, without the simultaneous incorporation of fundamental variables such as comorbidities, therapeutic intensity, and care context. Population-based evidence demonstrates that structural and health system factors impact cardiovascular outcomes more than isolated demographic variables.^3^

In this context, despite the observed clinical differences, the study does not delve into the analysis of the social determinants of health, limiting the interpretation of the findings.^4^ Furthermore, the data collection period (2013–2015) imposes restrictions on extrapolating the results to current clinical practice.

We commend the authors for their initiative and hope that their considerations will contribute to the scientific debate on racial inequalities and hypertension control.
