# Supplementary material for: Considerações Metodológicas sobre Disparidades Étnico-Raciais e Controle da Hipertensão Arterial
Source: Arq Bras Cardiol. 2026 Jun 23;123(5):e20260128. [Article in Portuguese] doi: 10.36660/abc.20260128 (PMC13399037; doi:10.36660/abc.20260128)
Supplement: Related texts

# Reply: Carta-resposta

**Authors:** Maicon Borges Euzebio, Weimar Kunz Sebba Barroso

**Affiliations:** 1 Instituto Federal de Goiás Águas Lindas de Goiás GO Brasil Instituto Federal de Goiás, Águas Lindas de Goiás, GO – Brasil; 2 Universidade Federal de Goiás Faculdade de Medicina Unidade de Hipertensão Goiânia GO Brasil Unidade de Hipertensão, Faculdade de Medicina, Universidade Federal de Goiás, Goiânia, GO – Brasil; 3 Universidade Federal de Goiás Faculdade de Medicina Goiânia GO Brasil Programa de Pós-Graduação em Ciências da Saúde, Faculdade de Medicina, Universidade Federal de Goiás, Goiânia, GO – Brasil; 4 Universidade Federal de Goiás Goiânia GO Brasil Universidade Federal de Goiás, Goiânia, GO – Brasil

**Prezado Editor,**

Agradecemos aos autores da correspondência pelo interesse em nosso artigo intitulado "Comparação dos Valores de Pressão Arterial e dos Medicamentos Anti-Hipertensivos Utilizados por Brasileiros não Afrodescendentes e Afrodescendentes com Hipertensão" e pelas reflexões apresentadas. Consideramos que o debate científico sobre desigualdades raciais no controle da hipertensão arterial é essencial para o avanço do conhecimento na área.

Inicialmente, em relação à definição operacional de raça/cor, reconhecemos que a classificação racial no Brasil apresenta elevada complexidade sociológica, histórica e biológica. Nesse sentido, a raça deve ser compreendida como uma construção social, que influencia o acesso a recursos, a exposição a fatores de risco e os desfechos em saúde.^1^

O presente estudo não teve como objetivo estabelecer critérios conceituais para a definição de afrodescendência, mas sim operacionalizar essa variável de forma metodologicamente consistente a partir dos dados disponíveis no Primeiro Registro Brasileiro de Hipertensão Arterial.^2^ Assim, adotou-se como grupo de interesse os indivíduos autodeclarados pretos, enquanto os demais participantes foram agrupados como não pretos.

Essa estratégia não teve como finalidade simplificar a complexidade da categorização racial no Brasil, mas assegurar consistência metodológica e coerência analítica. No contexto brasileiro, a classificação de raça/cor segue o padrão estabelecido pelo Instituto Brasileiro de Geografia e Estatística (IBGE),^3^ baseado na autodeclaração nas categorias branca, preta, parda, amarela e indígena. Conforme amplamente discutido na literatura e apresentado neste estudo, a agregação das categorias preta e parda é utilizada para compor a denominada população negra, refletindo não apenas aspectos fenotípicos, mas também processos históricos, sociais e estruturais que influenciam as desigualdades em saúde.^1^

A opção por analisar isoladamente a população autodeclarada preta fundamenta-se em evidências consistentes que demonstram maior vulnerabilidade social e piores indicadores de saúde nesse grupo, incluindo maior prevalência de hipertensão arterial, menor acesso aos serviços de saúde e piores desfechos clínicos.^1,4–7^ Essa abordagem permite maior sensibilidade na identificação de desigualdades, evitando a diluição de efeitos em agrupamentos mais amplos.

Adicionalmente, a escolha por indivíduos autodeclarados pretos encontra respaldo na literatura epidemiológica internacional, na qual essa população é mais consistentemente utilizada como representação de indivíduos afrodescendentes, favorecendo a comparabilidade entre estudos.^1^

No campo das ciências sociais e da saúde coletiva, é frequente a utilização de estratégias analíticas baseadas na dicotomização entre "brancos" e "não brancos", com o objetivo de evidenciar desigualdades estruturais. Estudos do Instituto de Pesquisa Econômica Aplicada demonstram a utilização dessa lógica como ferramenta analítica relevante para a compreensão das iniquidades sociais e raciais.^8^

De forma análoga, o presente estudo adotou a categorização entre "pretos" e "não pretos", considerando seu objetivo central de compreender as especificidades clínicas e terapêuticas na população preta. Tal estratégia não apenas aumenta a sensibilidade analítica, como também contribui para a visibilização de desigualdades historicamente negligenciadas, configurando-se como uma abordagem alinhada ao enfrentamento do racismo estrutural no campo científico.

À luz das recomendações contemporâneas para a comunicação científica, a descrição de indivíduos por ascendência regional — como africana — é considerada adequada quando fundamentada em pesquisas formais, sendo recomendada, sempre que possível, a especificação da origem geográfica.^9^ Nesse sentido, a utilização do termo "afrodescendente" no título do estudo foi intencional, visando alinhamento com a literatura internacional, enquanto a análise foi conduzida com base na autodeclaração como preto, conforme disponibilidade do banco de dados.

No que se refere à metodologia estatística, a escolha entre testes paramétricos e não paramétricos foi baseada na avaliação prévia da distribuição dos dados, por meio de testes de normalidade e inspeção gráfica, conforme descrito na metodologia.^1^

A verificação da distribuição dos dados constitui etapa essencial da análise estatística. O uso indiscriminado de testes paramétricos, frequentemente motivado por conveniência, é reconhecido como fonte de erro, podendo gerar inferências equivocadas quando suas premissas não são atendidas.^10^

Nesse contexto, a utilização de testes não paramétricos em situações de não normalidade representa uma escolha metodológica apropriada, pois esses métodos não dependem das mesmas premissas e reduzem o risco de conclusões incorretas.^10^ Ainda que apresentem menor poder estatístico, priorizam a validade das inferências.

Dessa forma, a escolha do teste estatístico baseada na distribuição dos dados não constitui decisão arbitrária ou exclusivamente post hoc, mas sim uma adequação técnica necessária para garantir rigor analítico.

Em relação aos modelos de regressão, a análise foi conduzida com base nas variáveis disponíveis no registro, não sendo possível incluir de forma padronizada variáveis como gravidade da doença ou determinantes sociais mais amplos, o que foi reconhecido como limitação do estudo.

O uso de betabloqueadores foi analisado como variável de interesse clínico em um contexto observacional, sem a intenção de estabelecer causalidade, mas de identificar padrões de utilização.

Quanto ao plano analítico, embora não formalmente descrito como pré-especificado, seguiu princípios epidemiológicos clássicos, incluindo análises descritivas, comparações bivariadas e regressões multivariadas.

Reconhecemos, ainda, o papel central dos determinantes sociais da saúde nas desigualdades cardiovasculares, incluindo fatores como renda, escolaridade e acesso aos serviços de saúde.^1,6,11^

Por fim, quanto ao período de coleta (2013–2015), reconhecemos limitações na extrapolação para a prática clínica atual. Ainda assim, os dados permanecem relevantes para a compreensão das desigualdades estruturais em saúde no Brasil.

Cumprimentamos os leitores pelas contribuições e reiteramos que o diálogo científico qualificado é fundamental para o avanço do conhecimento nessa área.

## References

[1] Euzébio MB, Vitorino PVO, Brandão AA, Barbosa ECD, Feitosa ADM, Malachias MVB (2025). Blood Pressure Control and Anthropometric Differences in Afro-Descendants and Other Ethnic Groups in Hypertensive Brazilian Populations. Glob Heart.

[2] Jardim PC, Souza WK, Lopes RD, Brandão AA, Malachias MV, Gomes MM (2016). I RBH - First Brazilian Hypertension Registry. Arq Bras Cardiol.

[3] Instituto Brasileiro de Geografia e Estatística (2023). Identificação Étnico-Racial da População, por Sexo e Idade: Resultados do Universo.

[4] Mendes PM, Nobre AA, Griep RH, Guimarães JMN, Juvanhol LL, Barreto SM (2018). Association between Perceived Racial Discrimination and Hypertension: Findings from the ELSA-Brasil Study. Cad Saude Publica.

[5] McEvoy JW, McCarthy CP, Bruno RM, Brouwers S, Canavan MD, Ceconi C (2024). 2024 ESC Guidelines for the Management of Elevated Blood Pressure and Hypertension: Developed by the Task Force on the Management of Elevated Blood Pressure and Hypertension of the European Society of Cardiology (ESC) and Endorsed by the European Society of Endocrinology (ESE) and the European Stroke Organisation (ESO). Eur Heart J.

[6] Akinyelure OP, Jaeger BC, Oparil S, Carson AP, Safford MM, Howard G (2023). Social Determinants of Health and Uncontrolled Blood Pressure in a National Cohort of Black and White US Adults: The REGARDS Study. Hypertension.

[7] Barroso WKS, Rodrigues CIS, Bortolotto LA, Mota-Gomes MA, Brandão AA, Feitosa ADM (2021). Brazilian Guidelines of Hypertension - 2020. Arq Bras Cardiol.

[8] Souza NBA, Oliveira JJA, Carvalho CC (2025). Desafios Contemporâneos e Serviço Social: Reflexões Críticas sobre Políticas Sociais e Capitalismo.

[9] Flanagin A, Frey T, Christiansen SL, AMA Manual of Style Committee (2021). Updated Guidance on the Reporting of Race and Ethnicity in Medical and Science Journals. JAMA.

[10] Nahm FS (2016). Nonparametric Statistical Tests for the Continuous Data: The Basic Concept and the Practical Use. Korean J Anesthesiol.

[11] Barroso WKS, Batista SR, Vitorino PVO, Sousa ALL (2022). Influence of Racial Composition on Blood Pressure Control in the Brazilian Population: The Need for New Perspectives Beyond Drug Treatment. Arq Bras Cardiol.

# Reply

**Authors:** Maicon Borges Euzebio, Weimar Kunz Sebba Barroso

**Affiliations:** 1 Águas Lindas de Goiás GO Brazil Instituto Federal de Goiás, Águas Lindas de Goiás, GO – Brazil; 2 Goiânia GO Brazil Unidade de Hipertensão, Faculdade de Medicina, Universidade Federal de Goiás, Goiânia, GO – Brazil; 3 Goiânia GO Brazil Programa de Pós-Graduação em Ciências da Saúde, Faculdade de Medicina, Universidade Federal de Goiás, Goiânia, GO – Brazil; 4 Goiânia GO Brazil Universidade Federal de Goiás, Goiânia, GO – Brazil

**Dear Editor,**

We thank the authors of the correspondence for their interest in our article entitled "Comparison of Blood Pressure Values and Antihypertensive Medications Used in Non-Afro-Descendant and Afro-Descendant Brazilians with Hypertension" and for the reflections presented. We believe that scientific debate on racial inequalities in the control of hypertension is essential for the advancement of knowledge in this area.

Initially, regarding the operational definition of race/color, we recognize that racial classification in Brazil presents a high degree of sociological, historical, and biological complexity. In this sense, race should be understood as a social construct that influences access to resources, exposure to risk factors, and health outcomes.^1^

The present study did not aim to establish conceptual criteria for the definition of Afro-descendants, but rather to operationalize this variable in a methodologically consistent manner based on data available in the First Brazilian Registry of Arterial Hypertension.^2^ Thus, individuals who self-identified as Black were adopted as the group of interest, while the other participants were grouped as non-Black.

This strategy was not intended to simplify the complexity of racial categorization in Brazil, but to ensure methodological consistency and analytical coherence. In the Brazilian context, race/color classification follows the pattern established by the Brazilian Institute of Geography and Statistics (IBGE),^3^ based on self-declaration in the categories white, black, brown, yellow, and indigenous. As widely discussed in the literature and presented in this study, the aggregation of the black and brown categories is used to compose the so-called black population, reflecting not only phenotypic aspects, but also historical, social, and structural processes that influence health inequalities.^1^

The decision to analyze the self-declared Black population in isolation is based on consistent evidence demonstrating greater social vulnerability and worse health indicators in this group, including a higher prevalence of hypertension, less access to health services, and worse clinical outcomes^1,4–7^ This approach allows for greater sensitivity in identifying inequalities, avoiding the dilution of effects in broader groupings.

Additionally, the choice of self-declared black individuals is supported by international epidemiological literature, in which this population is more consistently used as a representation of Afro-descendant individuals, favoring comparability between studies.^1^

In the fields of social sciences and public health, analytical strategies based on the dichotomy between "whites" and "non-whites" are frequently used to highlight structural inequalities. Studies by the Institute for Applied Economic Research demonstrate the use of this logic as a relevant analytical tool for understanding social and racial inequities.^8^

Similarly, the present study adopted the categorization between "black" and "non-black," considering its central objective of understanding the clinical and therapeutic specificities in the black population. This strategy not only increases analytical sensitivity but also contributes to making visible historically neglected inequalities, thus configuring itself as an approach aligned with confronting structural racism in the scientific field.

In light of contemporary recommendations for scientific communication, describing individuals by regional ancestry—such as African—is considered appropriate when based on formal research, and specifying geographic origin is recommended whenever possible.^9^ In this sense, the use of the term "Afro-descendant" in the study title was intentional, aiming for alignment with international literature, while the analysis was conducted based on self-declaration as Black, according to database availability.

About statistical methodology, the choice between parametric and non-parametric tests was based on a prior assessment of the data distribution, using normality tests and graphical inspection, as described in methodology.^1^

Checking the distribution of data is an essential step in statistical analysis. The indiscriminate use of parametric tests, often motivated by convenience, is recognized as a source of error and can generate erroneous inferences when their premises are not met.^10^

In this context, the use of non-parametric tests in situations of non-normality represents an appropriate methodological choice, since these methods do not depend on the same assumptions and reduce the risk of incorrect conclusions.^10^ Although they have lower statistical power, they prioritize the validity of inferences.

Therefore, choosing a statistical test based on the distribution of the data is not an arbitrary or purely post hoc decision, but rather a necessary technical adjustment to ensure analytical rigor.

Regarding the regression models, the analysis was conducted based on the variables available in the registry, and it was not possible to include variables such as disease severity or broader social determinants in a standardized way, which was recognized as a limitation of the study.

The use of beta-blockers was analyzed as a variable of clinical interest in an observational context, without the intention of establishing causality, but rather to identify patterns of use.

Regarding the analytical plan, although not formally described as pre-specified, it followed classic epidemiological principles, including descriptive analyses, bivariate comparisons, and multivariate regressions.

We also recognize the central role of social determinants of health in cardiovascular inequalities, including factors such as income, education, and access to health services^1,6,11^

Finally, regarding the data collection period (2013–2015), we acknowledge limitations in extrapolating the data to current clinical practice. Nevertheless, the data remain relevant for understanding structural health inequalities in Brazil.

We commend the readers for their contributions and reiterate that qualified scientific dialogue is fundamental to the advancement of knowledge in this area.
